# Efficacy and Conflicts of Interest in Randomized Controlled Trials Evaluating Headspace and Calm Apps: Systematic Review

**DOI:** 10.2196/40924

**Published:** 2022-09-20

**Authors:** Alison O'Daffer, Susannah F Colt, Akash R Wasil, Nancy Lau

**Affiliations:** 1 Palliative Care and Resilience Research Program Center for Clinical and Translational Research Seattle Children's Research Institute Seattle, WA United States; 2 Perelman School of Medicine University of Pennsylvania Philadelphia, PA United States; 3 Department of Psychology University of Pennsylvania Philadelphia, PA United States; 4 Department of Psychiatry and Behavioral Sciences University of Washington Seattle, WA United States

**Keywords:** mHealth, psychological interventions, mobile health, mental health, health applications, health apps, mindfulness, meditation app, digital health application, digital health intervention

## Abstract

**Background:**

Although there are thousands of mental health apps, 2 apps, Headspace and Calm, claim a large percentage of the marketplace. These two mindfulness and meditation apps have reached tens of millions of active users. To guide consumers, clinicians, and researchers, we performed a systematic review of randomized controlled trials (RCTs) of Headspace and Calm.

**Objective:**

Our study aimed to evaluate intervention efficacy, risk of bias, and conflicts of interest (COIs) in the evidence base for Headspace and Calm, the two most popular mental health apps at the time of our search.

**Methods:**

To identify studies, we searched academic databases (Google Scholar, MEDLINE, and PsycINFO) and the websites of Headspace and Calm in May 2021 for RCTs of Headspace and Calm testing efficacy via original data collection, published in English in peer-reviewed journals. For each study, we coded (1) study characteristics (eg, participants, sample size, and outcome measures), (2) intervention characteristics (eg, free vs paid version of the app and intended frequency of app usage), (3) all study outcomes, (4) Cochrane risk of bias variables, and (5) COI variables (eg, presence or absence of a preregistration and the presence or absence of a COI statement involving the company).

**Results:**

We identified 14 RCTs of Headspace and 1 RCT of Calm. Overall, 93% (13/14) of RCTs of Headspace and 100% (1/1) of RCTs of Calm recruited participants from a nonclinical population. Studies commonly measured mindfulness, well-being, stress, depressive symptoms, and anxiety symptoms. Headspace use improved depression in 75% of studies that evaluated it as an outcome. Findings were mixed for mindfulness, well-being, stress, and anxiety, but at least 40% of studies showed improvement for each of these outcomes. Studies were generally underpowered to detect “small” or “medium” effect sizes. Furthermore, 50% (7/14) of RCTs of Headspace and 0% (0/1) of RCTs of Calm reported a COI that involved Headspace or Calm (the companies). The most common COI was the app company providing premium app access for free for participants, and notably, 14% (2/14) of RCTs of Headspace reported Headspace employee involvement in study design, execution, and data analysis. Only 36% (5/14) of RCTs of Headspace were preregistered, and the 1 RCT of Calm was not preregistered.

**Conclusions:**

The empirical research on Headspace appears promising, whereas there is an absence of randomized trials on Calm. Limitations of this study include an inability to compare Headspace and Calm owing to the dearth of RCTs studying Calm and the reliance on author reports to evaluate COIs. When determining whether or not mental health apps are of high quality, identification of high-quality apps and evaluation of their effectiveness and investigators’ COIs should be ensured.

## Introduction

### Background

Mental health problems are leading contributors to the global burden of disease [1]. As a result, efforts to improve population-level mental health and wellness are a public health priority. Although empirically supported treatments exist for mental health problems, most people in need of support do not access traditional mental health treatments [2]. Common barriers to treatment access include high costs, low supply and availability of clinicians, stigma toward professional treatments, and preferences for self-help [3,4].

Mental health help-seekers have gravitated toward low-barrier, cost-effective prevention and intervention programs, mainly mental health apps. Although there are thousands of mental health apps, data through 2021 have shown that 2 apps, Calm and Headspace, are the most popular and consistently rank the highest in the number of downloads and user activity [5-10]. Both apps include mindfulness meditation and deep breathing content and allow users the ability to select the topic (eg, sleep or stress relief), length, and modality of a guided sessions each time they use the app (with the option to follow specific modules in order). The app landscape is dynamic, but 2019 estimates suggest that each app reaches approximately 5-9 million monthly active users, and the apps are responsible for approximately 90% of total monthly active users [5,11]. Given the widespread dissemination of these apps, evaluation of the quality of the evidence for these apps is a public health priority. Such a review could help identify if, for whom, and for which conditions these mental health apps have been shown to be effective. Although previous systematic reviews and meta-analyses have shown that mental health apps can be effective for depression and anxiety [7,12-14], there is little overlap between the apps that are evaluated in academic research [15] and those that are widely disseminated on public-facing app stores [5,16]. Thus, reviewing Headspace and Calm is an important priority, and the findings from existing reviews of mental health apps may not generalize to these commercially popular apps.

Headspace Inc and Calm are both for-profit companies, and both companies use research findings to promote their products. Increasing interest in the clinical robustness of these apps [17] presents a potential conflict of interest (COI): companies may have incentives to publish “positive” findings and suppress negative or inconclusive results. Even among academic researchers, incentives to publish “positive” results has contributed to biased literature, leading to concerns about the reproducibility of psychological science [15]. There has also been a growing conversation about “researcher degrees of freedom”— decisions in data collection and analysis that may contribute to the elevated rate of false positives in psychological science [18]. While these concerns are always worth considering when reviewing academic literature, they may be especially salient when for-profit companies are performing or funding research on their own products (eg, elevated estimates of the effectiveness of antidepressant medications [19]). It is plausible that similar concerns could be present in digital mental health interventions [20], especially in cases where companies are explicitly funding, sponsoring, or participating in clinical trials.

### Objectives

In this study, we systematically reviewed randomized controlled trials (RCTs) of Headspace and Calm, the two most popular mental health apps. These two apps dominate the mental health app market, both in absolute terms (reaching millions of users each month) and relative terms (reaching up to 90% of mental health app users). We aimed to (1) evaluate the efficacy of these apps and (2) evaluate the risk of bias and COIs in the studies contributing to this evidence base. Owing to a wide range of outcomes of interest across studies, we did not conduct a meta-analysis. The purpose of this review is to provide researchers, clinicians, and consumers with up-to-date information regarding the evidence base, risk of bias, and COIs of the two most popular mental health apps.

## Methods

### Search Strategy

Our approach is outlined in detail in the PRISMA (Preferred Reporting Items for Systematic Reviews and Meta-Analyses) diagram (Multimedia Appendix 1). Two authors (AO and SC) conducted a literature review via Google Scholar, MEDLINE, and PsycINFO databases using the search terms “*[app name]”* AND “*smartphone”* in May 2021 to identify peer reviewed RCTs of Headspace and Calm. To supplement this procedure, we also identified articles via the websites for Headspace and Calm, which list peer-reviewed publications on their respective apps. The date range for this search had no start date and ended in May 2021. Inclusion criteria were as follows: RCTs of Headspace and Calm testing efficacy, published in peer reviewed journals, in English only, and solely including original data collection. Exclusion criteria included non-Headspace or -Calm papers non-RCTs, nonoriginal data collection, conference abstracts, or student theses, non–English-language papers, and papers not published in a peer reviewed journal.

Three authors (SC, AO, and NL) retrieved and independently reviewed the full text of all eligible studies. Two authors (AO and NL) coded half of the included articles, and one author (SC) coded all the articles, such that each article was coded by at least 2 coders. To resolve discrepancies, coders conducted consensus conversations and referred to the articles for resolution.

### Data Extraction

#### Trial Outcomes

We extracted the following information from each included article: participants, sample size, intervention adherence, treatment condition, and type of control condition. We extracted all reported outcomes, regardless of whether the outcome in question was examined as a primary, secondary, or exploratory outcome, and we documented whether each outcome measured was positive or negative (or null). A positive outcome was defined as the intervention condition outperforming the control with statistically significant findings. A negative outcome was defined as the control condition outperforming the intervention with statistically significant findings. A null outcome was defined as nonsignificant differences between the intervention and control conditions. To better characterize the studies and to understand how participants engaged with the apps, we also examined additional variables: (1) whether or not users had access to the premium (paid) versions of the app, (2) whether users were instructed to use specific parts of the app, or if they were told to use the app freely and choose which content to access, (3) the intended frequency of use, (4) the actual of frequency of use (if measured), (5) the length of the intervention (eg, 4 weeks), and (6) incentives that were provided to participants for their participation in the study.

Power calculations were performed using G*Power (version 3.1) assuming an α of .05 and a desired power of at least 0.823) [21]. We considered a study’s power as “high” if the study included enough participants to detect a between-group standardized mean difference of 0.3, “medium” if it included enough participants to detect a standardized mean difference of 0.5, and “low” if it did not include enough participants to detect a standardized mean difference of 0.5. Thus, studies with over 278 participants were coded as “high,” studies with between 102 and 278 participants were coded as “medium,” and those with fewer than 102 participants were coded as “low” in power.

#### Risk of Bias

We assessed risk of bias using the Cochrane collaboration’s risk of bias tool. Three authors (NL, SC, and AO) independently assessed risk of bias by applying 7 criteria from the Cochrane collaboration’s tool for assessing risk of bias: (1) evaluating random sequence generation (selection bias), (2) allocation concealment (selection bias), (3) blinding of participants and personnel (performance bias), (4) blinding of outcome assessment (detection bias), (5) incomplete outcome data (attrition bias), and (6) selective reporting [22].

#### Assessment of COIs

There have been concerns about the reproducibility in psychological science [15], which may be especially salient when research is conducted or supported by profit-driven companies [19]. Thus, it is important to apply additional codes to assess study rigor and bias, going beyond those included in the Cochrane framework. To develop these additional codes, we reviewed relevant work on risk of bias disclosure recommendations [15,22] and open science practices [23,24]. Specifically, we examined if (1) the companies had any role in the study, (2) the companies initiated the study, (3) the companies were involved in analyzing data, (4) the companies provided funding for the study, (5) the companies were mentioned in the acknowledgments section, (6) members of the companies were included as coauthors, and (7) trial preregistration. Preregistration—the act of specifying research questions, relevant variables, and planned analyses before data collection—is a highly valued open science practice that is thought to reduce the use of questionable research practices [25].

## Results

### Study Details

#### Overview

Our final sample consisted of 15 studies. We identified 14 RCTs of Headspace [26-39] and 1 RCT of Calm [40]. Table 1 summarizes the characteristics and findings of each study on all outcomes measured. We categorized specific study outcomes into 5 overarching constructs (mindfulness, psychological well-being, stress, anxiety, and depression) representative of the psychosocial outcomes that mental health apps including Headspace and Calm purport to target. This categorization scheme enabled us to descriptively synthesize the various outcome measures into overarching domains.

**Table 1:** Included studies (N=15).

Authors (year; country)			Participants (sample size)		Intervention		Control group		Adherence		Positive results		Null or inconclusive results		Gender (% female) and age (mean)
**Calm**															
	Huberty et al (2019; United States) [40]	College students (n=88)		10 minutes of daily use for 8 weeks		Waitlist		On average, intervention participants completed 37.9/70 (54%) minutes of meditation per week over the course of the study		Improved stress, mindfulness, and self-compassion		N/A^a^		88 Intervention: 20.41 years; Control: 21.85 years	
**Headspace**															
	Bennike et al (2017; Denmark) [26]	University staff novice meditators (n=95)		10 minutes daily for week 1, 15 minutes daily for week 2, and 20 minutes daily for week 3		Cognitive-training app use for 30 days		On average, intervention participants completed 302.7/450 (67%) minutes of the required meditation minutes over the study period		Improved dispositional mindfulness and mind wandering		N/A		69% in the intervention group; 71% in the control group Intervention: 41.4 years; control: 43.4 years	
	Bjorkstrand et al (2019; Sweden) [27]	Adults without extensive meditation experience (n=26)		Daily 10-20–minute guided mindfulness meditation sessions over 4 weeks		Waitlist		On average, intervention participants completed 13.2 minutes of meditation per day		Improved retention of extinction learning on day 2		No effect on fear acquisition or extinction of conditioned response on day 1		79% (86% in the intervention group; 73% int he control group) 35.1 years (intervention: 35.6 years; control: 34.5 years)	
	Bostock et al (2019; United Kingdom) [28]	Adult employees of 2 firms in the United Kingdom reporting work stress (n=238)		45 sessions of guided mindfulness meditation over 8 weeks		Waitlist		On average, participants completed 16.6/45 sessions (37%)		Improved global well-being, daily positive affect, anxiety and depressive symptoms, job strain, and workplace social support		Marginally significant improvement in systolic blood pressure. No effect on diastolic blood pressure		59% 35.5 years	
	Champion et al (2018; United Kingdom) [29]	Adult novice meditators (n=74)		Daily use for 30 days		Waitlist		On average, intervention participants completed 6.21/10 (62%) sessions in the first 10 days and 11.66/20 (58%) sessions in the second 20 days^b^		Improved satisfaction with life, stress, and resilience		N/A		55% 39.4 years	
	DeSteno et al (2018; United States) [30]	College student novice meditators (n=46)		Daily meditation training (approximately 15 min) for 3 weeks		Daily logic problem		53/77 (68%) intervention participants completed all required sessions		Improved aggression		No effect on anger or executive control		Gender % not reported Age range: 18-24 years (average not reported)	
	Economides et al (2018; United States) [31]	Adult novice meditators (n=88)		10 sessions in 1 month		Mindfulness or meditation psychoeducational audiobook		69/88 (78%) participants completed all sessions		Improved irritability, affect, and stress from external issues		No effect on stress from internal pressure		57% 28% aged 18-24 years; 26% aged 25-29 years, 27% aged 30-39 years, and 19% aged 40-49 years	
	Flett et al (2018; New Zealand) [32]	College students (n=208)		Daily use for 10 days		Intervention arm 2: Smiling Mind app use; control: Evernote app use		On average, intervention participants completed 8.24/10 sessions (82%)^b^		Improved depressive symptoms and college adjustment (for both Headspace and Smiling Mind users). Improved mindfulness for Headspace users. (Improved resilience for Smiling Mind users). Improvements were maintained for participants who continued to use intervention apps		No differences in flourishing, stress, or anxiety. No effect on resilience for Headspace users. (No effect on mindfulness for Smiling Mind users)		Gender % not reported 20.08 years	
	Howells et al (2016; United Kingdom) [33]	Adult app users (n=121)		10 minutes daily for 10 days		List-making app use (Catch Notes)		Not reported		Improved positive affect and depressive symptoms		No effect on satisfaction with life, flourishing, or negative affect		87% 40.7 years	
	Kubo et al (2019; United States) [34]	Arm 1: patients with a diagnosis of cancer (n=72). Arm 2: their caregivers (26)		8 weeks of daily mindfulness sessions delivered via Headspace app		Waitlist		Not reported		Patients: improved overall well-being. (Caregivers: improved FFMQ^c^ observing mindfulness domain score)		Patients: no statistically significant differences in change in anxiety, depression, sleep, or fatigue		Arm 1: 69% Arm 2: 58% Mean age not reported	
	Lim et al (2015; United States) [35]	College student novice meditators (n=56)		14 sessions plus daily quiz over 3 weeks		14 sessions of cognitive-training app plus daily questionnaire		Not reported		Improved compassionate responding		No effect on empathic accuracy		54% 19.4 years	
	Noone and Hogan (2018; Ireland) [36]	College students (n=91)		30 mindfulness meditation sessions over 6 weeks		30 sham meditations delivered through Headspace interface		On average, intervention participants completed 15/30 (50%) sessions		N/A		No difference between groups in mindful disposition, critical thinking, or executive functioning		76% 20.92 years	
	Quinones and Griffiths (2019; United Kingdom) [37]	Adult novice meditators with signs of compulsive internet use (n=994)		Daily 10-minute mindfulness podcast		Active control: muscle relaxation podcast. Passive control: waitlist		Not reported		Improved mindfulness and compulsive internet use in the intervention group compared to active control and waitlist groups		No differences between mindfulness and active control groups in anxiety or depression, but both outperformed waitlist group		Intervention group: 38%; active control: 42%; waitlist control: 37% Intervention group: 39 years; active control group: 40 years; waitlist control: 41 years	
	Rosen et al (2018; United States) [38]	Women diagnosed with breast cancer (n=112)		Self-guided app-delivered mindfulness training for 8 weeks		Waitlist		On average, intervention patients used the app 18/72 (25%) days.		Improved quality of life and mindfulness.		N/A		100% Intervention group: 51.4 years; control group: 53.22 years	
	Yang et al (2018; United States) [39]	Medical students (n=88)		App-delivered mindfulness training over 30 days		Waitlist		On average, intervention participants completed 11.97/30 (40%) sessions^b^		Improved well-being and stress		No differences between groups for mindfulness		64% 25.11 years	

^a^N/A: not applicable.

^b^Self-report data.

^c^FFMQ: Five Facet Mindfulness Questionnaire

#### RCTs of the Headspace App

Among the RCTs of the Headspace app, 43% (6/14) of studies recruited novice meditators (individuals with some experience with meditation practices prior to the study), 29% (4/14) of them included college students, 14% (2/14) of them included patients with cancer, and 7% (1/14) of them included individuals with compulsive internet use. In other words, most studies focused on samples from the general population, rather than individuals with elevated levels of depression, anxiety, or another mental disorders. Overall, 50% (7/14) of studies included a measure of mindfulness, 57% (8/14) of them measured well-being, 36% (5/14) of them measured stress, 29% (4/14) of them measured depressive symptoms, and 29% (4/14) of them measured anxiety symptoms. Furthermore, 43% (6/14) of the studies used waitlist control conditions, 43% (6/14) of them had active control conditions, and 14% (2/14) of them had both an active and a waitlist control condition. Of the 5 RCTs with only active control conditions, 33% (2/6) of studies used cognitive training apps, 17% (1/6) of studies had participants do daily logic problems, 17% (1/6) of studies used a mindfulness or meditation psychoeducation audiobook, and 17% (1/6) of studies had participants complete sham meditation sessions through the Headspace app.

In 93% (13/14) of studies, participants were allowed to access content from the premium (paid) version of the app. In 93% (13/14) of studies, participants were instructed to use specific parts of the app (as opposed to navigating the app freely). The intervention period ranged from 14 days to 70 days (mean 33.14, SD 14.53 days). In 79% (11/14) of studies, participants were instructed to use the app for at least 10 minutes each day. Overall, 29% (4/14) of studies did not report app adherence data, 21% (3/14) of studies asked participants for self-reported usage data, and 50% (7/14) of studies used backend app usage data from Headspace to evaluate app usage. App adherence metrics varied greatly (including days of meditation completed, minutes of meditation completed, number of participants who completed entire intervention, and number of completed sessions), and these data are provided in Multimedia Appendix 2. No paper reported lower than 25% adherence or higher than 90.16% adherence on the measure they utilized. Furthermore, 71% (10/14) of studies offered some sort of incentive to participants (eg, gift card, course credit, premium app access, and lottery entry) and 29% (4/14) of studies offered no incentive for participation.

#### RCT of the Calm App

The 1 RCT of the Calm app recruited college students, who were not required to have a mental health diagnosis or clinically significant distress. Of our 5 outcome domains of interest, the Calm RCT measured stress and mindfulness. The active control condition was a waitlist control. Participants were allowed to access content from the premium (paid) version of the app and were instructed to use specific modules within the app. Participants were instructed to use the app daily for at least 10 minutes a day for 8 weeks. On average, intervention participants completed 37.9 out of 70 minutes (54%) of meditation per week over the course of the study. Participants were given gift cards as an incentive for completing questionnaires.

### Trial Outcomes

#### RCTs of the Headspace App

**Figure 1:**
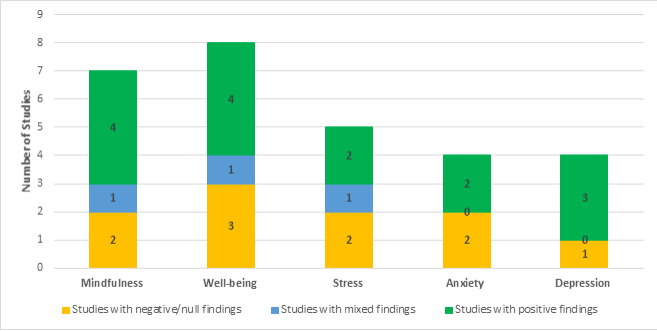
Summary of findings from randomized controlled trials (RCTs) of Headspace. “Mixed findings” refers to when 2 or more measures were used to evaluate the same outcome domain in an RCT and these measures yielded conflicting results.

Figure 1 presents a summary of main findings from the 14 RCTs of Headspace. We categorized findings in the 5 domains of interest as “positive” (ie, the intervention group showed significant improvement compared to the control group), “mixed” (ie, 2 or more measures of the same outcome domain that yielded conflicting results), or “null” (ie, the intervention group did not outperform the control group) for each outcome domain. Of the RCTs of Headspace that evaluated mindfulness, 57% (4/7) had positive findings, 14% (1/7) had mixed findings, and 29% (2/7) had null findings. Of the RCTs of Headspace that evaluated well-being, 50% (4/8) had positive findings, 13% (1/8) had mixed findings, and 38% (3/8) had null findings. For the RCTs of Headspace that evaluated stress, 40% (2/5) had positive findings, 20% (1/5) had mixed findings, and 40% (2/5) had null findings. For anxiety, 50% (2/4) of studies had positive findings, and 50% (2/4) had null findings. Finally, for RCTs of Headspace evaluating depression, 75% (3/4) had positive findings and 25% (1/4) had null findings. We were unable to calculate effect size pooled estimates owing to the small number of studies, the variability in outcome measures, and the wide range of timing for administration of postassessments.

Sample sizes (number of participants included in analyses) ranged from 46 to 994 (mean 174, median 102, SD 234). Applying our coding system, 64% (9/14) of the studies had low power (<102 participants), 29% (4/14) studies had medium power (between 102 and 278 participants), and 7% (1/14) of studies had high power (>278 participants). More specifics on outcomes (including additional outcomes from each RCT) are provided in Multimedia Appendix 2.

#### RCT of the Calm App

In the single RCT of the Calm app, participants in the intervention arm showed significantly improved stress and mindfulness scores than those in the control arm. In total, 88 participants were included in the analyses, and the study had low power (<102 participants).

### Risk of Bias

#### RCTs of the Headspace App

Overall, 100% (14/14) of studies were judged as having a low risk of bias on two of the Cochrane criteria: *random sequence generation* and *allocation concealment*. On the *blinding of* *participants and personnel* domain and the *blinding of outcome assessment* domain, 50% (7/14) studies received a rating of low risk and 50% (7/14) received a rating of high risk. On the *incomplete outcome* *data* domain, 57% (8/14) of studies received a rating of low risk and 43% (6/14) received a rating of high risk. Finally, on the *selective reporting* domain, 29% (4/14) of studies received a rating of low risk and 71% (10/14) of studies received a rating of unclear. These 10 studies were not preregistered, so we could not determine if the authors engaged in selective reporting of outcomes. For the *other bias* category, 79% (11/14) of studies were rated as having a low risk, 14% (2/14) of them were rated as having a high risk, and 7% (1/14) of them were rated as unclear. Itemized Cochrane risk of bias results can be found in Multimedia Appendix 2.

#### RCT of the Calm App

The singular RCT of the Calm app was judged as having a low risk of bias on the *random sequence generation* and *allocation concealment* domains*.* On the *blinding of participants and personnel* and *blinding of outcome assessment* domains*,* it received a high risk rating. *Incomplete outcome data* was rated as having a low risk of bias and *selective reporting* was rated as having a high risk of bias. The RCT of Calm had a low risk of bias for the *other bias* category. Itemized Cochrane risk of bias results can be found in Multimedia Appendix 2.

### Assessment of COIs

In addition to the standard Cochrane risk of bias categories, we evaluated preregistration and COIs related to the involvement of app companies in the 15 RCTs identified.

#### RCTs of the Headspace App

##### Preregistration

Of the 14 RCTs of Headspace, only 36% (5/14) were preregistered.

##### Assessment of COIs

**Figure 2:**
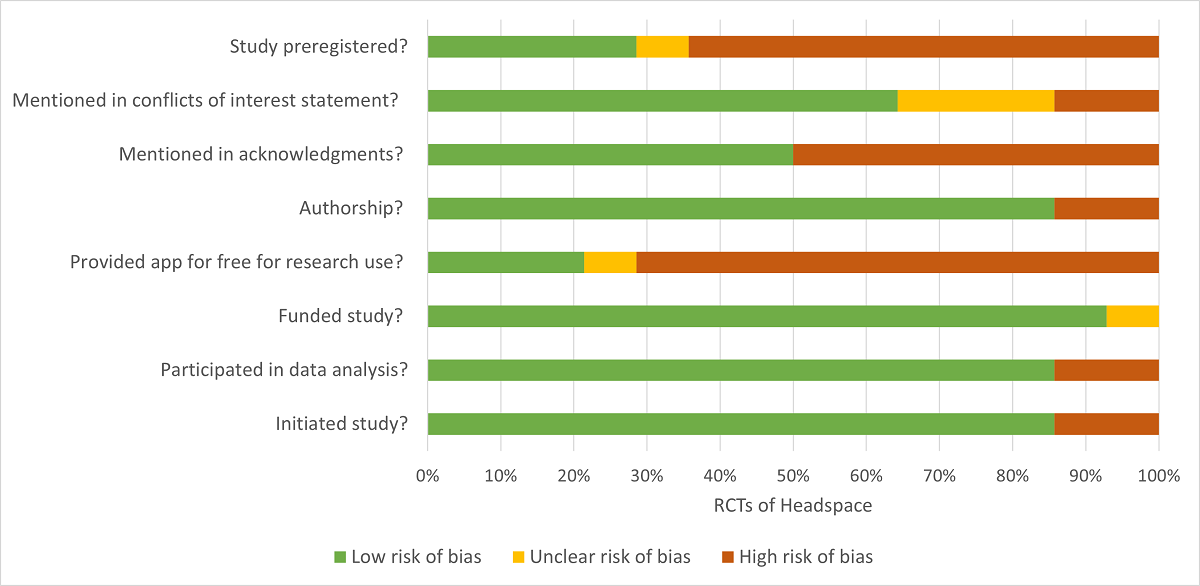
Evaluation of conflicts of interest in randomized controlled trials (RCTs) of Headspace.

Half of the studies (7/14) mentioned a COI in their COI statement that involved Headspace or Calm, 21% (3/14) of them did not include a COI statement, and 29% (4/14) of them explicitly stated that there was no COI. In 14% (2/14) of studies, individuals from Headspace or Calm were involved in the study’s conception or execution. Overall, 93% (13/14) of studies were not funded by Headspace, and for 7% (1/14) of studies, it was unclear whether the app company had funded the study. In 14% (2/14) of studies, individuals from Headspace or Calm were involved in data analysis and were included as coauthors. In 71% (10/14) of studies, Headspace Inc provided premium app access at no cost to researchers for participants to use, 21% (3/14) of studies did not use complimentary access from Headspace, and for 7% (1/14) of studies, this usage was unclear. Figure 2 depicts the COI data of these studies.

#### RCT of the Calm App

##### Preregistration

The 1 RCT of the Calm app was not preregistered.

##### Assessment of COIs

For the 1 RCT of the Calm app, Calm (the company) was not involved in initiating the study, data analysis, study funding, or authorship. Researchers did not specify in the paper whether Calm provided app use free of charge for this study. The company was not mentioned in the acknowledgments or COI statements.

## Discussion

### Principal Findings

We performed a systematic review of RCTs evaluating Headspace and Calm, the two most popular mental health apps. First, we evaluated the efficacy of Calm and Headspace. For Calm, additional RCTs will be needed before the question of efficacy can be addressed empirically. For Headspace, our review of RCTs demonstrated that the efficacy findings are inconclusive. RCTs of Headspace showed that Headspace use reliably improved depression. Findings were mixed for mindfulness, well-being, stress, and anxiety, but at least 40% of studies for each of these 4 outcomes showed improvement from the intervention. The studies mostly focused on members of the general population; we found that relatively few studies have examined the efficacy of these apps with clinical samples. Most studies were not powered to detect “small” or “medium” effects. App adherence data were measured inconsistently. Second, our review characterized the risk of bias and COIs in the available evidence. For all studies, lack of preregistration was a main concern for risk of bias. Direct app company involvement in authorship and study procedures was low for both apps. For Headspace papers, the provision of free use of the premium version of the app was another key finding. The single RCT evaluating Calm did not find COIs with regard to the company’s involvement of study conduct, analysis, or authorship.

### Comparison to Prior Work

Our general discussion of studies in aggregate mainly refer to Headspace owing to the limited number of RCTs identified for Calm. Despite the mixed findings and underpowered studies, we believe that the evidence supporting an intervention should be considered in light of its costs. Even if only a small proportion of individuals who use a mental health app experience symptom reduction attributable to the app, this “small proportion” could include millions of individuals who would not have accessed other forms of evidence-based support [41,42]. Furthermore, users who do not benefit from the apps can discontinue using them with low opportunity costs. Headspace and Calm are unguided self-help apps with relatively lower costs than other kinds of mental health promotion interventions (eg, psychotherapy, medications, and professional coaching). Both Headspace and Calm offer a free version of the app, and the premium versions cost US $13 per month and US $15 per month, respectively (or both offer an annual plan for US $70 per year), which are considerably more affordable than traditional mental health interventions [42] (eg, US $60-$250 per session for private-pay psychotherapy [43]).

Notably, there are several ways in which app usage in RCTs may differ from app usage in naturalistic settings. The trials included in this review focused on college students, healthy volunteers from the general population, and novice meditators. In most of the trials, users were instructed to access specific content within the apps. In contrast, when apps are used in naturalistic settings, users are free to choose the content that they want to access. Additionally, engagement with apps tends to be higher in trials, as investigators can promote engagement through financial incentives, and participants in research trials may feel committed to participating fully in the study [44]. Thus, findings from randomized trials may not fully generalize to app usage in real-world settings. We were unable to draw conclusions on app adherence data owing to variability in measurement. App adherence is a crucial component of understanding the real-world validity of mobile health (mHealth) interventions; hence, adoption of standardized reporting tools is necessary for appropriate evaluation of adherence in future systematic reviews on mHealth interventions [45].

Given the low cost of Headspace, the fact that multiple randomized trials have supported its effectiveness in some samples and individuals who do not benefit from Headspace can discontinue its use with a low opportunity cost, Headspace may be a promising intervention. More evidence on Calm is needed. The funding that Headspace provides promotes the acquisition of empirical evidence on mental health apps, which is positive, but the provision of app use free of charge for research presents several relevant risks for bias. First, there is evidence of a higher potential for bias when people who work at a for-profit company are involved in study design, conduct, and analysis [46]. Second, researchers who are interested in studying psychological interventions or constructs including mindfulness will be more likely to study a mindfulness app being offered free of charge [7]. Other apps that may be equally or more effective may not be able to financially support research in this way. This difference accounts for the imbalance between Headspace and Calm with respect to the number of published RCTs we found in our review. The resulting plethora of research on one app in comparison to other mental health apps may lead consumers to believe that that app is the “best” intervention or the most evidence based, when the lack of studies on other mental health apps is potentially attributable to financial inaccessibility.

### Future Directions

Our review demonstrates several gaps to be addressed by future research on popular mental health apps. First, future research could examine for whom these apps are effective, and how much of the intervention someone must complete to achieve desired positive effects. Precision mental health techniques could be used to identify individuals who are most likely to respond to apps, minimum intervention time, and what content might be most helpful for a given individual.

Future randomized trials of mental health apps could also evaluate the effectiveness of apps when users are instructed to use the app freely rather than when they are instructed to access specific preselected modules within the app, particularly in naturalistic settings. For example, Headspace gifted free app access to educators during the COVID-19 pandemic [47], and future similar circumstances could provide an opportunity to study app efficacy and engagement.

The variability in outcomes across RCTs prevented us from calculating effect sizes or other statistics in these data, limiting our ability to draw conclusions. Future work could attempt to standardize patient-reported outcomes in clinical trials on mental health apps to enable future comparisons, especially via meta-analysis.

The involvement of app companies in the research process introduces a risk of bias in studies evaluating mental health apps. We recommend that when evaluating an existing intervention that is provided free of charge, researchers should use an active control to demonstrate how Headspace and Calm perform in comparison with alternative apps. With the goal of improving mental health outcomes for users, strategies should be explored to increase the number of open access apps available for research.

### Limitations

There are a few important limitations to our study. First, the app market is dynamic, and new apps may increase in popularity rapidly or over time. This study is time bound, since our search was conducted in May 2021 when Headspace and Calm were the most downloaded and widely used mental health apps. This may change by the time of or after publication of this review. Second, this review was not preregistered, and a protocol was not published ahead of time, thus potentially increasing the risk of bias in our review. Third, the disparity in the number of RCTs for Headspace compared to those of Calm limited our ability to investigate the Calm app thoroughly. We were not able to directly compare efficacy and COI variables between Headspace and Calm owing to only finding 1 RCT of Calm. Since the number of RCTs for Headspace and Calm was beyond our control, we discussed the results without comparing the two apps and encouraged additional RCTs on Calm. Fourth, we only captured risk of bias and COI information based on what authors reported; hence, we may not be aware of all potential COIs. Fifth, we reported results on the basis of significance, but significant findings do not necessarily mean that improvements in psychosocial outcomes are clinically significant, and we did not evaluate the data with respect to clinically meaningful differences.

### Conclusions

The wide adoption of apps including Headspace and Calm provides an opportunity to address population-level mental health. We hope that this review inspires further work on mental health apps, both adding to the current evidence base on Headspace and Calm and evaluating other mental health apps. We advise clinicians, researchers, and consumers of clinical research to ask similar questions about COIs when consuming research, particularly research evaluating products from for-profit companies using science-based marketing to promote their product. Once a product such as Headspace or Calm is widely used, it can be easily accepted on face value as effective, but we want to inspire other researchers to evaluate the nuances of the evidence base, especially since popular mental health apps are already reaching millions of people each month. If effective apps disseminate widely, they may play an extremely important role in improving mental health and wellness worldwide.

## Appendix

Multimedia Appendix 1 PRISMA (Preferred Reporting Items for Systematic Reviews and Meta-Analyses) Diagram.

Multimedia Appendix 2 Cochrane Risk of Bias Summary.

## Abbreviations

COI: conflict of interest
mHealth: mobile health
PRISMA: Preferred Reporting Items for Systematic Reviews and Meta-Analyses
RCT: randomized controlled trial
