# Supplementary figures and images for: Efficacy and Conflicts of Interest in Randomized Controlled Trials Evaluating Headspace and Calm Apps: Systematic Review
Source: JMIR Ment Health. 2022 Sep 20;9(9):e40924. doi: 10.2196/40924 (PMC9533203; doi:10.2196/40924)

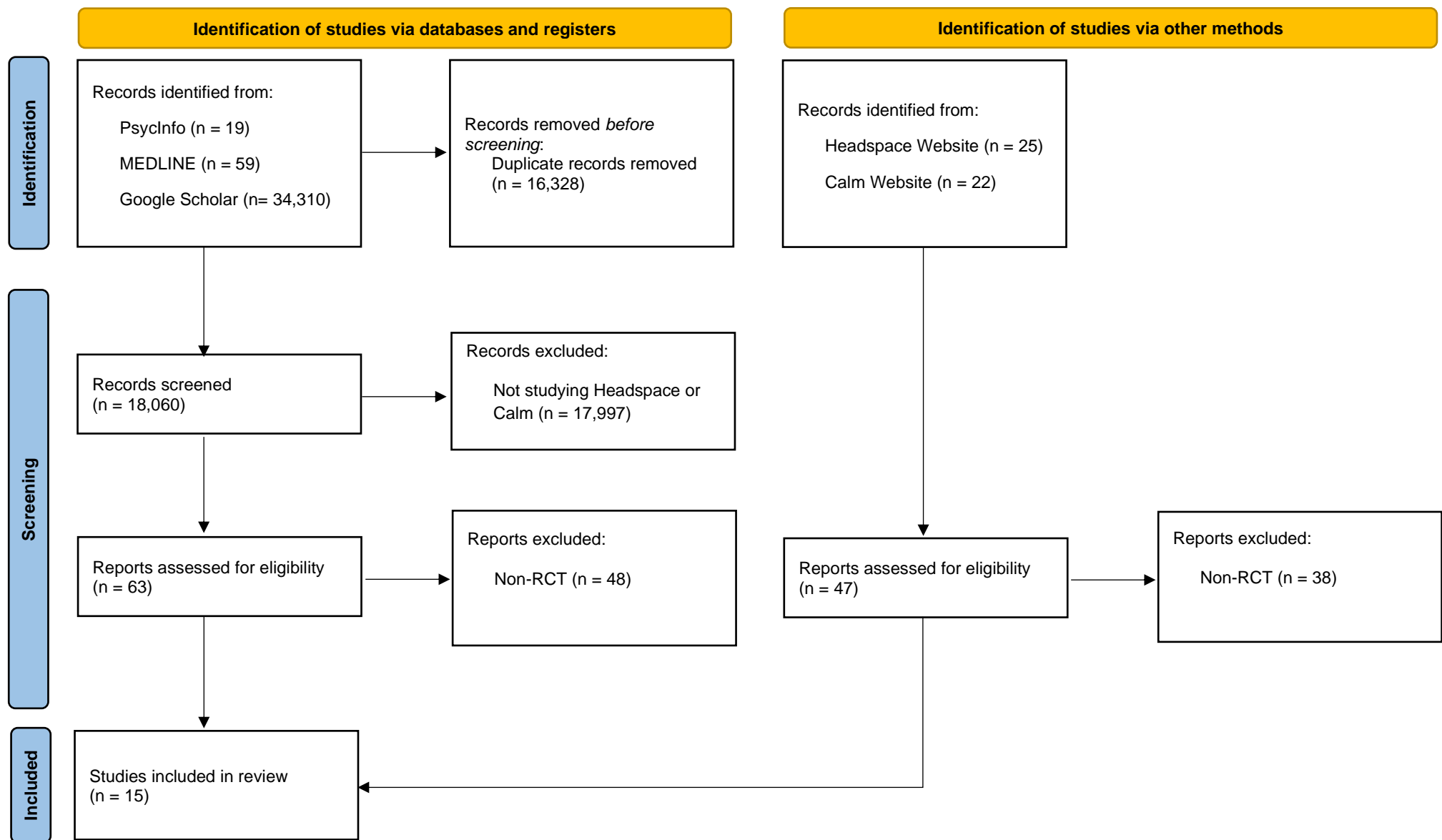

Supplement: Multimedia Appendix 1 [file mental_v9i9e40924_app1.pdf]

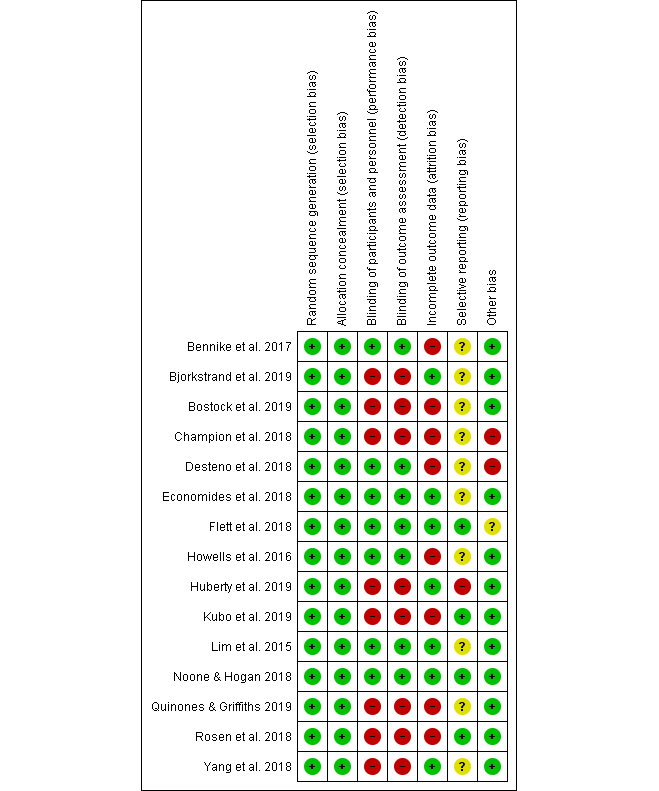

Supplement: Multimedia Appendix 2 [file mental_v9i9e40924_app2.png]
